# An integrated machine learning framework for cerebral aneurysm rupture status classification using clinical, morphological, and hemodynamic features

**DOI:** 10.1371/journal.pone.0360923

**Published:** 2026-10-30

**Authors:** Mostafa Zakeri, Amirhossein Atef, Mohammad Aziznia, Azadeh Jafari

**Affiliations:** School of Mechanical Engineering, College of Engineering, University of Tehran, Tehran, Iran; Universidade da Beira Interior, PORTUGAL

## Abstract

Accurate assessment of cerebral aneurysm rupture status remains a critical challenge in neurovascular research due to the complex interplay between geometric, hemodynamic, and clinical factors. In this study, we developed and evaluated a machine-learning framework for retrospective rupture-status classification in 708 patient-specific aneurysms by integrating 55 predictors: three clinical variables, 35 morphological descriptors, and 17 hemodynamic parameters derived from computational fluid dynamics (CFD) simulations. Blood flow was modeled as incompressible laminar flow with a non-Newtonian Carreau–Yasuda viscosity model to capture shear-thinning behavior in cerebral arteries. We compared 13 supervised classifiers, 10 unsupervised clustering approaches, and two neural-network models. Model training and hyperparameter optimization were performed using stratified five-fold cross-validation within a 90/10 training–testing split, with performance evaluated using ROC–AUC, precision, recall, specificity, and F1-score. Among the evaluated models, BaggingSVM achieved the strongest discrimination on the independent test set (ROC–AUC = 0.78), followed by SVM (0.75) and Gaussian Process (0.75). A scenario comparison using BaggingSVM showed that the combined feature set improved classification performance (AUC = 0.78) compared with clinical+morphological features (AUC = 0.76) and clinical+hemodynamical features (AUC = 0.71) settings. Sensitivity-oriented threshold tuning increased recall for ruptured aneurysm detection, reaching values up to 0.96 for BaggingSVM. Permutation feature importance analysis revealed consistent dominance of ellipticity index, low shear area ratio (LSAR), aneurysm location, irregularity and aneurysm angle across models. a small subset of variables contributes disproportionately to rupture-status classification, although the reduced-feature analysis indicates that lower-ranked predictors collectively provide additional discriminatory information. The proposed framework demonstrates the value of integrating physiologically informed hemodynamic modeling with machine learning to improve data-driven rupture status assessment and provides a scalable foundation for future clinical decision-support studies in cerebrovascular diagnostics.

## Introduction

Cerebral aneurysm is a largely asymptomatic cerebrovascular condition that affects between 2 and 5 percent of the world’s population [1] and predominantly occurs in the proximal arterial bifurcations in the circle of Willis [2]. Diagnostic methods for assessing aneurysms encompass a range of imaging modalities, including magnetic resonance (MR) and computed tomography (CT), each offering its own set of advantages and disadvantages [3,4]. Different treatment options are available, each associated with specific benefits and challenges. For example, coil embolization demonstrated that aneurysm sac thickness plays a dominant role in deformation and stress reduction, with up to 70% stress decrease observed after coiling [5]. Despite advances in treatment and imaging technologies, aneurysm rupture remains a major clinical concern due to its association with aneurysmal subarachnoid hemorrhage (aSAH), which carries high mortality and long-term neurological disability rates [6].

Formation, growth, and rupture of cerebral aneurysms can be attributed to various factors, including genetic and environmental factors [7], morphological and clinical factors [8], and hemodynamic factors [9]. For example, a diameter-to-parent-vessel ratio greater than 2.3 (morphological factor) [10], occurring in the anterior communicating artery (ACA) (clinical factor) [11], containing complex unstable flow patterns [12] and lower wall shear stress (WSS) (hemodynamic factor) [13] are more susceptible to rupture. In addition, geometric characteristics of the surrounding vasculature, including vessel diameter and bifurcation angles, have also been shown to influence aneurysm development and rupture behavior [14]. In a comprehensive study on the contribution of morphological features in rupture risk assessment, ellipticity index, size ratio, and irregularity were introduced as dominant features, respectively [15]. Moreover, in a thorough study on the rupture classification of cerebral aneurysms based on hemodynamic parameters, the low shear area ratio, relative residence time, and turnover time were recognized as the most important hemodynamic parameters [16]. Recent computational and experimental studies further highlight the importance of WSS-related metrics, oscillatory shear index and other flow-related forces in aneurysm wall remodeling and rupture vulnerability [17].

Geometric characteristics of the aneurysm and surrounding vasculature can substantially affect local hemodynamics. Recent CFD studies of stent-induced vessel deformation have demonstrated corresponding changes in intra-aneurysmal flow velocity, WSS, and oscillatory shear index (OSI) [18,19]. Given the relatively small dimensions and pulsatile flow conditions of cerebral arteries, non-Newtonian effects can become important, particularly in low-shear regions within aneurysms. Accordingly, non-Newtonian rheological models can provide a more physiologically representative description of cerebral aneurysm hemodynamics [20–22]. Notably, the Carreau-Yasuda model [23] is widely embraced for its ability to rectify the over-prediction of WSS by the Newtonian model and accurately represent the shear-thinning behavior at lower strain rates [24]. This model aligns well with experimental data on blood behavior across different strain rates [25] and demonstrates commendable performance in simulating pulsatile flows [26]. Furthermore, mechanical loading conditions can significantly alter intraluminal pressure and stress distribution, even when rupture thresholds are not exceeded [27], emphasizing the need for accurate hemodynamic modeling in rupture risk analysis. Recent computational studies have further demonstrated differences in WSS, time-averaged WSS, OSI, and residence-time-related measures between ruptured and unruptured aneurysms, reinforcing the potential value of combining multiple hemodynamic descriptors rather than relying on a single flow metric [28]. Because rupture status reflects the combined influence of clinical, morphological, and hemodynamic factors, evaluating these domains separately may overlook complementary information that emerges when they are considered jointly. This motivates the use of machine-learning approaches capable of integrating heterogeneous predictors within a common framework.

Machine-learning (ML) models have increasingly been applied to cerebral aneurysm rupture-status classification because they can capture nonlinear relationships among multiple interacting predictors. Previous studies have evaluated conventional classifiers, ensemble methods, and deep-learning approaches, generally reporting moderate to strong discrimination depending on the cohort, feature set, and validation strategy [29–36]. Systematic reviews nevertheless identify persistent challenges related to dataset heterogeneity, generalizability, and model interpretability [36–38]. Several studies have also demonstrated promising results. For example, Tanioka et al. [39] achieved an accuracy of 0.78 by leveraging hemodynamic and morphological features from a dataset of 226 patients. In another investigation involving 615 patients, Random Forest (RF) models achieved an AUC of 0.81 [40]. However, cerebral aneurysm data are inherently sensitive, and metrics such as recall and precision are also important and informative [41]. In this context, a study involving 124 patients achieved results with a precision of 0.80 and recall of 0.82 incerebral aneurysm rupture classification [42]. Moreover, in our previous studies, we trained ML models for rupture classification, one based primarily on morphological parameters and another on hemodynamic parameters, achieving accuracy and precision of approximately 0.80 and a recall greater than 0.90 in each study [15,16]. Despite these advances, the incremental contribution of morphological and hemodynamic information and the stability of feature importance when these domains are evaluated jointly under the same modeling framework remain insufficiently established.

The central aim of the present study is therefore not simply to merge previously reported parameters, but to evaluate rupture status classification within a unified framework that simultaneously incorporates clinical, morphological, and hemodynamic descriptors and compare the results of combined framework with each set of parameters in isolation. This integrated analysis enables direct comparison of feature importance across domains and clarifies which predictors remain robust when all available information is considered together. Accordingly, this study develops an integrated machine-learning framework for retrospective cerebral aneurysm rupture status classification using 55 predictors derived from clinical variables, aneurysm morphology, and CFD-based hemodynamic. Beyond model performance, we focused on integrating clinical, morphological, and hemodynamic information would improve retrospective rupture-status discrimination relative to clinical + morphological and clinical + hemodynamic feature configurations, and that a subset of cross-domain predictors would emerge as consistently influential when all feature domains were evaluated jointly.

## Methodology

### Study Population

This study did not involve human participants, animals, or any identifiable personal data. Furthermore, no experiments or procedures were conducted that fall under institutional or national ethics review guidelines.

We analyzed 708 patient-specific cerebral aneurysm geometries from the AneuX morphology database, an open-access, multi-centric database combining data from three European projects: AneuX project (www.aneux.ch), @neurIST project (www.aneurist.org) and Aneurisk (http://ecm2.mathcs.emory.edu/aneuriskweb/index) (https://zenodo.org/records/6678442), including 253 ruptured and 455 unruptured cases from Sheffield, Milan, Geneva, and Barcelona [43,44]. Rupture status was obtained directly from the clinical metadata supplied with the AneuX database and was not independently assigned or reclassified by the authors of the present study. Fig 1 summarizes the cohort by aneurysm location within the Circle of Willis and by patient sex. We categorized aneurysm locations into six anatomical regions: internal carotid artery (ICA), middle cerebral artery (MCA), anterior communicating artery (ACA), basilar/vertebral artery (BA/VA), posterior communicating artery (PCA), and superior cerebellar artery (SCA). The bar chart in Fig 1 shows the ICA was the most common location (323/708), followed by MCA (181/708) and ACA (143/708). The database was female-predominant (520 females vs. 188 males), and the number of female cases exceeded male cases across all anatomical locations.

**Fig 1:**
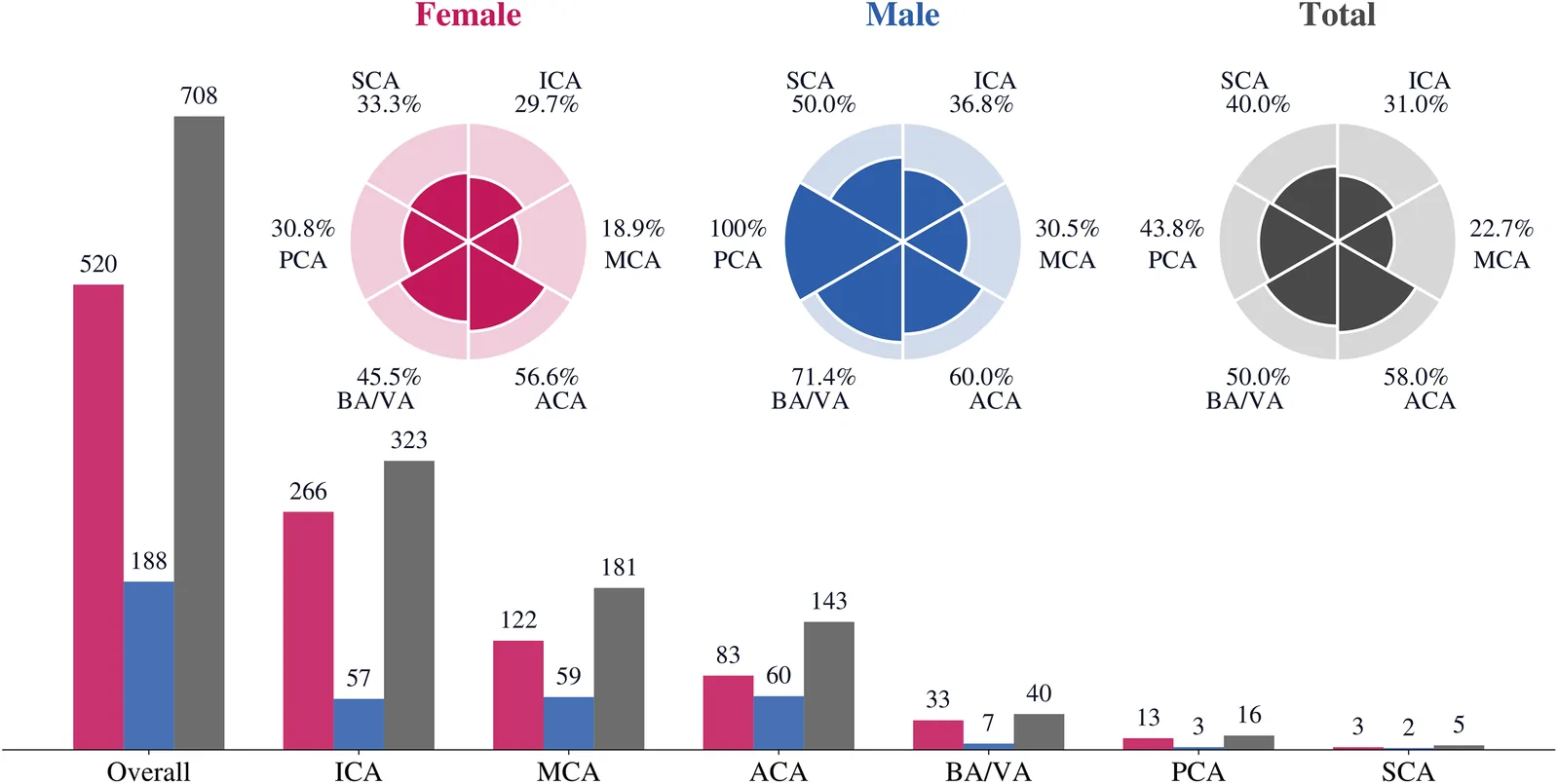
Distribution of aneurysm cases by anatomical location and sex with corresponding rupture proportions. Bar charts display the total number of patients by location (Overall, ICA, MCA, ACA, BA/VA, PCA, SCA) stratified by Female, Male, and Total cases. Radial plots illustrate rupture proportions for each location separately for females, males, and the combined cohort. Within each radial slice, the pale sector represents the full set of cases at that location, while the solid inner region represents ruptured cases, with its area proportional to the rupture percentage. Percent values are displayed outside each slice.

Moreover, radial plots in Fig 1 further report rupture proportions by location and sex. Overall, ACA showed the highest rupture proportion (58% across both sexes; 56.6% among females). Among males, PCA showed the highest rupture proportion (100%); however, this estimate is based on only three male PCA cases and should be interpreted cautiously. In contrast, MCA exhibited the lowest rupture proportion across categories (18.9% in females, 30.5% in males, and 22.7% overall), despite being the second most frequent aneurysm location in the dataset.

### Clinical and morphological parameters

Three of the considered parameters in this study are clinical variables: age, sex, and the location of the aneurysm within the circle of Willis, which were numerically encoded for model input: sex (Male = 0, Female = 1), aneurysm location (MCA = 0, ICA = 1, ACA = 2, PCA = 3, VA/BA = 4, SCA = 5). We extracted 35 aneurysm morphology features to quantify geometric characteristics associated with rupture status. Some parameters were already available in the database [44] (e.g., dome volume/area and several standard size/shape indices), and we directly used the provided values. For the remaining parameters, we performed measurement and calculation using Materialise 3-matic v13.0, ParaView v5.11.0, and mathematical relations, to ensure a consistent and complete feature set across all geometries. These parameters include: dome volume (DV), dome area (DA), maximum diameter (MD), central perpendicular height (CPH), neck diameter (ND), neck area (NA), aspect ratio (AR), bottleneck ratio (BR), diameter of the parent vessel at the inlet (DPVI), neck ratio (NR), size ratio (SR), maximum perpendicular height (MPH), irregularity (I), lateral or bifurcation status (LB), neck to dome ratio (NDR), volume to the ostium area (VOA), shape factor (SF), inflow angle (IMDA), aneurysm angle (MDNA), neck angle (INA), projection length (PL), ellipticity index (EI), non-sphericity shape index (NSI), undulation index (UI), maximum diameter parallel to the neck (MDPN), projection ratio (PR), conicity parameter (CP). In addition to established parameters, we included eight descriptors previously introduced in our morphology-focused study [15]: neck circumference (NC), ideal roundness (IR), ideal roundness ratio (IRR), ideal sphericity (IS), ideal sphericity ratio (ISR), outflow number (ON), cumulative outlet diameter (COD), and inlet-to-outlet(s) ratio (IOR). These descriptors were designed to capture geometric characteristics not fully represented by conventional indices. NC, IR, and IRR characterize neck geometry and deviation from a circular shape; IS and ISR characterize the deviation of the aneurysm sac from an ideal sphere of equivalent volume; and ON, COD, and IOR characterize the number and relative dimensions of outlet vessels. Complete mathematical definitions and calculation procedures are provided in Table A in S1 Appendix. Fig 2 presents representative examples of lateral and bifurcation aneurysms with both regular and irregular morphologies. For model input in the ML stage, we encoded categorical morphological descriptors using binary mapping (e.g., irregularity: regular = 0 vs irregular = 1; lateral/bifurcation: lateral = 0 vs bifurcation = 1).

**Fig 2:**
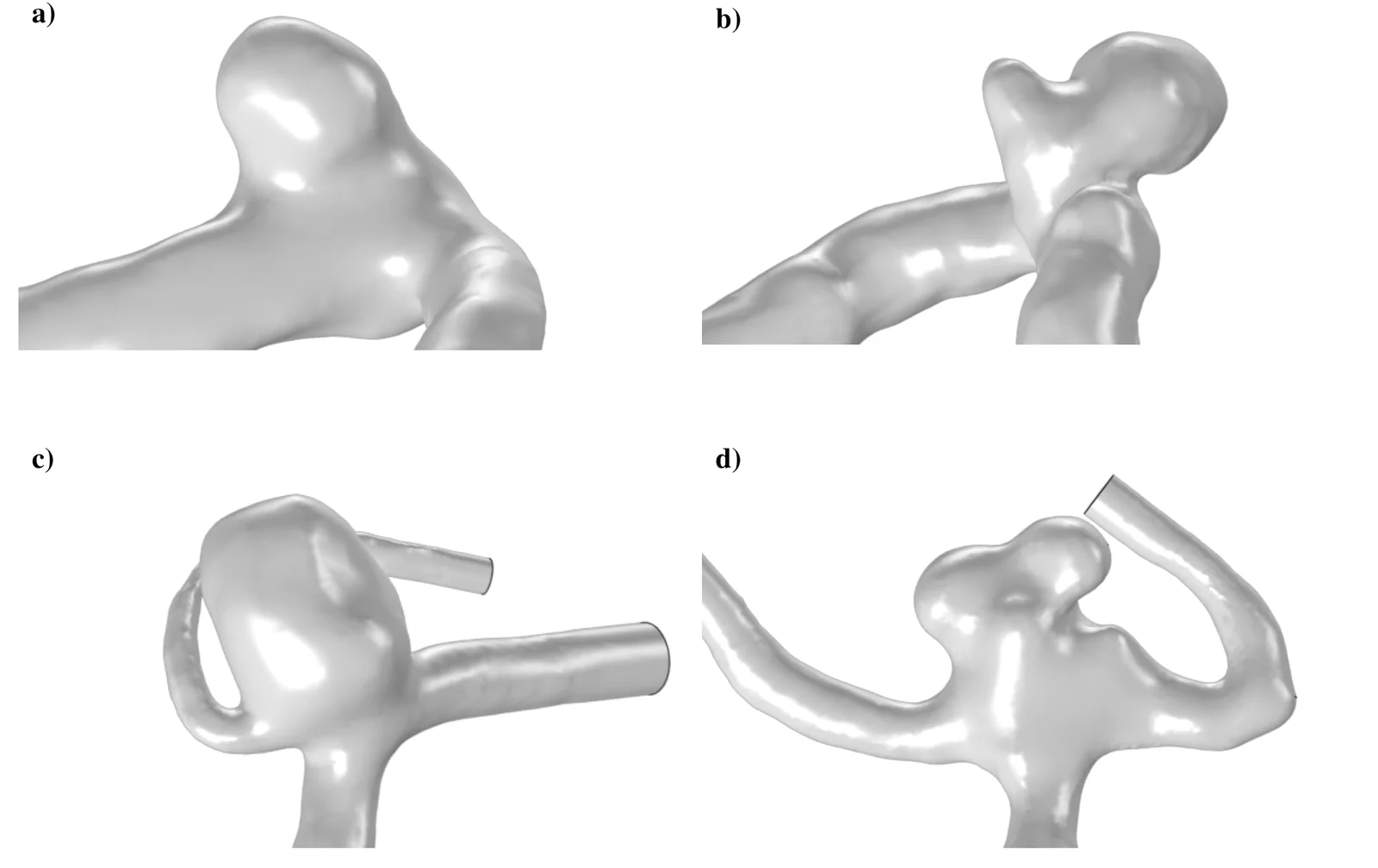
Examples of aneurysm morphology illustrating lateral and bifurcation types with regular and irregular characteristics. The panels show (a) lateral–regular (case ID: p447d), (b) lateral–irregular (case ID: p447a), (c) bifurcation–regular (case ID: p449), and (d) bifurcation–irregular (case ID: p482) configurations. The images were generated by the authors from the preprocessed three-dimensional vessel geometries used in this study. The vessel models were trimmed around the aneurysm region during preprocessing to emphasize sac morphology. These renderings are original to the present study and are not reproduced or adapted from previously published figures. The source geometries were obtained from the AneuX database [43].

For transparency and reproducibility, we provide the complete mathematical definitions and formulas for all morphological parameters, including the eight novel parameters, in S1 Appendix.

### Blood Flow Simulation and Hemodynamic Parameters

We performed patient-specific blood-flow simulations to obtain the hemodynamic parameter values used as inputs to our rupture-classification models. We first preprocessed each vascular geometry to make it suitable for CFD by removing imaging artifacts and improving surface quality (e.g., smoothing rough surfaces and sharp corners). We completed this preprocessing using vascular modeling toolkit (VMTK) (http://www.vmtk.org/download/) and Materialise 3-matic v13.0, and then we ran blood-flow simulations in COMSOL Multiphysics v6.0 for each aneurysm geometry.

We modeled blood flow as incompressible laminar flow, consistent with the range of Reynolds numbers (~300–1000) expected across cases due to differences in vessel diameter and flow velocity. We solved the incompressible continuity and Navier–Stokes momentum equations:


$$\begin{array}{c} \nabla \cdot \vec{V}=\text{0} \end{array}$$ (1)

$$\begin{array}{c} \rho \frac{D\vec{V}}{Dt}=-\nabla p+\nabla \cdot (\eta \left(\nabla \vec{V}\right)+{\left(\nabla \vec{V}\right)}^{T}) \end{array}$$ (2)

where $\vec{V}$ and p are the velocity and pressure fields of the fluid, ρ is density, t is time and η is dynamic viscosity. We implemented the Carreau–Yasuda shear-thinning viscosity model to enhance the robustness of the simulation results by accounting for the shear-thinning behavior of blood flow, as follows:


$$\begin{array}{c} \eta =\eta_{\infty }+\left(\eta_{\text{0}}-\eta_{\infty }\right){\left[\text{1}+{\left(\lambda {\left|{II}_{\dot{\gamma }}\right|}^{\frac{\text{1}}{\text{2}}}\right)}^{a}\right]}^{\frac{n-\text{1}}{a}} \end{array}$$ (3)

where $\eta_{\infty }$ and $\eta_{\text{0}}$ are dynamics viscosities at infinite and at zero shear rates, respectively. Besides, λ is the time constant, ${II}_{\dot{\gamma }}$ is the second invariant of the strain rate, a is the Yasuda coefficient, and n is the power-law index. We used the following parameters for blood flow: $\eta_{\text{0}}=\text{0}.\text{0}\text{5}\text{6}\;Pa.s$, $\eta_{\infty }=\;\text{0}.\text{0}\text{0}\text{3}\text{4}\text{5}\;Pa.s$, λ=1.902 s, n=0.22, and a = 1.25 [45].

Because inlet flow is pulsatile, we ran time-dependent simulations and used an algebraic multigrid (AMG) solver with the GMRES method. We applied a velocity inlet condition, no-slip at the vessel wall, and open boundary conditions at outlets. To improve numerical stability at startup, we initialized the simulation using a step function that increased from zero until reaching the minimum of the periodic waveform. The time step was determined automatically by COMSOL to satisfy the Courant–Friedrichs–Lewy (CFL) condition. We reported results from the second cardiac cycle to reduce any first-cycle startup artifacts; differences between the second and subsequent cycles were negligible, with a reported maximum WSS error at systolic peak of 0.3%. We used a representative cerebral artery velocity waveform and scaled it using patient-relevant parameters (age, sex, and Circle of Willis location) via systolic-peak velocities (Fig 3 and Table II in Zakeri et. al [16].).

**Fig 3:**
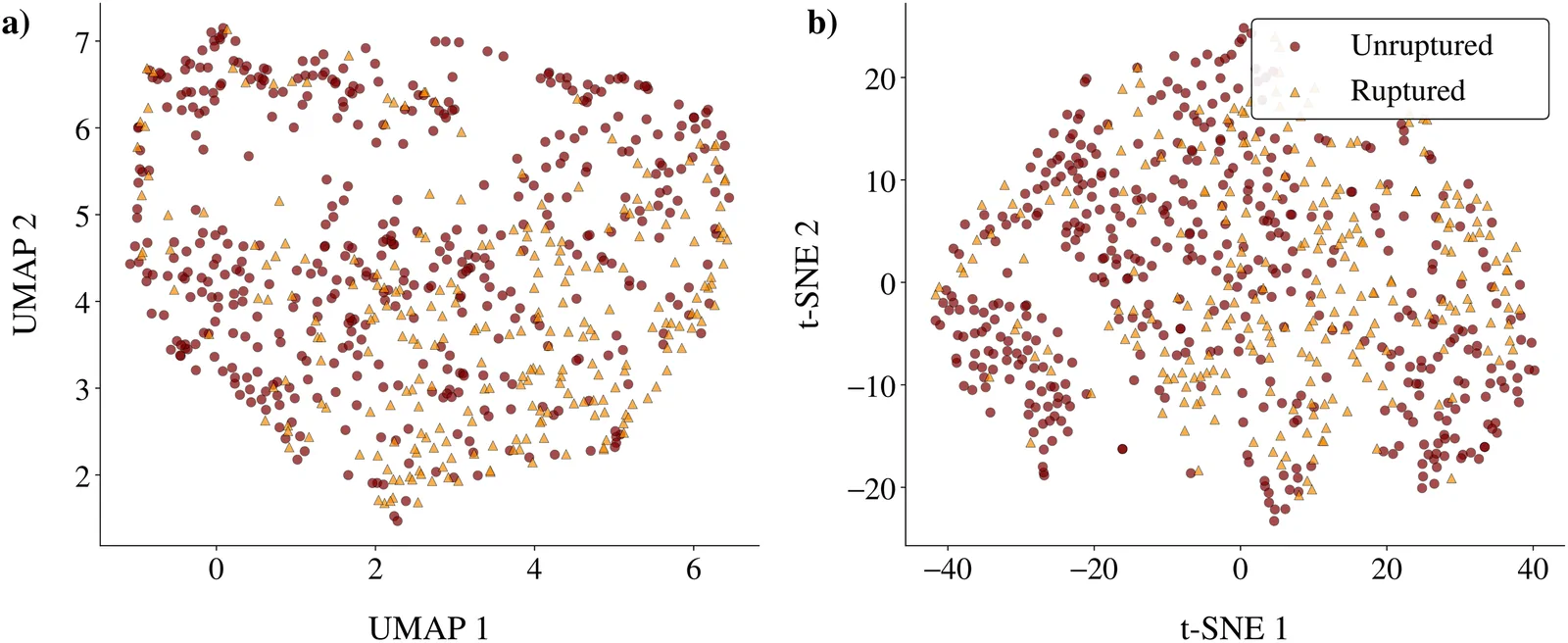
(a) UMAP and (b) t-SNE embeddings of standardized features, colored by rupture status. Each point represents an individual case.

We generated tetrahedral meshes in COMSOL and refined the mesh near the vessel wall because many of our hemodynamic metrics are wall-derived. We applied a five-layer boundary-layer mesh (stretching factor 1.2, automatic thickness, thickness adjustment factor 2, and corner refinement). To assess mesh independence, three refinement levels were evaluated using a refinement factor of 1.68: approximately 101,041 elements for the coarse mesh, 169,821 for the medium mesh, and 284,672 for the fine mesh. Average aneurysm-dome velocity and average aneurysm-wall WSS over the cardiac cycle were compared between refinement levels, and the medium mesh provided results comparable to the fine mesh while reducing computational cost. This standardized mesh-independence procedure was repeated for at least five different aneurysm geometries. The purpose of this analysis was to verify the robustness of the common meshing protocol rather than to perform case-specific mesh-independence testing for all 708 geometries. The same custom COMSOL mesh settings, including near-wall refinement and a five-layer boundary-layer mesh, were subsequently applied throughout the dataset.

From the simulation results, we extracted the hemodynamic variables used in our hemodynamic-focused work [16] and commonly in this study: wall shear stress (WSS), normalized wall shear stress (NWSS), wall shear stress gradient (WSSG), wall shear stress divergence (WSSD), time-averaged wall shear stress (TAWSS), oscillatory shear index (OSI), aneurysm formation indicator (AFI), relative residence time (RRT), low shear area ratio (LSAR), oscillatory velocity index (OVI), flow velocity (FV), pressure loss coefficient (PLC), energy loss (EL), turnover time (TT), normalized pressure difference (NPD), flow stability (FS), and flow complexity (FC). Mathematical definitions are provided in S1 Appendix, with corresponding visualizations in S1–S11 Figs and S1 Movie.

### Machine Learning

To explore the intrinsic structure of the feature space, nonlinear dimensionality reduction techniques were applied (Fig 3). UMAP (Uniform Manifold Approximation and Projection) and t-SNE (t-distributed Stochastic Neighbor Embedding) project high-dimensional features into a two-dimensional space while preserving neighborhood relationships between cases. UMAP aims to retain both local and partial global geometric structure of the data manifold, providing a representation that reflects broader feature organization. In contrast, t-SNE prioritizes preservation of local similarity, emphasizing small-scale neighborhood clustering while potentially distorting global distances. In both embeddings, ruptured and unruptured aneurysms are visualized as distinct point distributions, enabling qualitative assessment of class separation within the learned feature space. These projections are intended for exploratory visualization and do not represent a predictive model or classification outcome. This visualization suggests that the class boundary may be nonlinear, motivating the use of nonlinear classifiers.

We initially explored dimensionality reduction using Singular Value Decomposition (SVD) and Principal Component Analysis (PCA) to identify dominant variance directions in the feature space. However, restricting the model inputs to the top 10 principal components resulted in reduced classification performance compared to using all 55 original features. Since computational cost remained manageable, all supervised models were ultimately trained using the full feature set.

We used a two-stage evaluation strategy to prevent optimistic bias. First, the dataset was split into training and testing partitions using a stratified split (90% training, 10% testing). The stratified random split was selected instead of center-based hold-out validation because the contributing centers differed substantially in sample size and in the distributions of aneurysm location, sex, age, and rupture status. All model selection, hyperparameter tuning, and preprocessing were performed exclusively within the training set. The held-out test set was used only once for final performance reporting. Within the training set, models were tuned using stratified 5-fold cross-validation. Hyperparameters were selected to maximize AUC (primary) while monitoring recall and precision due to the clinical cost of false negatives. All input variables were standardized using parameters computed exclusively from the training set; the same parameters were then applied to the held-out test set to prevent data leakage. This was necessary for scale-sensitive models such as SVM and Gaussian Process classifiers.

In our comparison of various machine learning algorithms, we trained 10 unsupervised models, 13 supervised models, and 2 neural network models. This extensive approach allows us to address both classification and clustering concepts. The unsupervised algorithms used with their hyperparameters are reported in Table 1. These algorithms can identify similarities between disparate features without prior knowledge of the rupture status, thereby clustering the features into two principal categories. Since we were already aware of the rupture status of each case, we proceeded to identify each cluster based on its affinity with pre-existing labels related to rupture status. Finally, best-mapped accuracy was computed by optimally aligning cluster labels to ground-truth rupture labels using the Hungarian assignment algorithm to maximize agreement between clusters and true classes.

**Table 1:** Hyperparameter configuration of unsupervised clustering and density-based algorithms used for clustering of aneurysm cases.

Model	Hyperparameters
Nearest Neighbors	n_neighbors=9, radius=0.1, algorithm=‘auto’
K-Means	n_clusters = 2
Agglomerative Clustering	n_clusters = 2
Mean Shift	default (MeanShift())
Affinity Propagation	damping = 0.9999
Birch	threshold=100000, n_clusters=2
OPTICS	eps=1, min_samples=50
Spectral Clustering	n_clusters = 2
Gaussian Mixture	n_components = 2
DBSCAN	eps=500000, min_samples=3

We evaluated multiple supervised classifiers spanning linear, kernel-based, tree-based, ensemble, probabilistic, and neural network methods. In addition, a 1-Dimensional Convolutional Neural Network (1D-CNN) was implemented to model nonlinear interactions across morphological features. We trained the 1D CNN with a sigmoid activation to produce a continuous rupture probability p(y=1∣x) ∈ [0,1] as a non-binary classifier.


$$\begin{array}{c} \hat{p}=\sigma (z)=\frac{\text{1}}{\text{1}+e^{-z}} \end{array}$$ (4)

This output is interpreted as a model-estimated rupture likelihood under the empirical distribution of the dataset. All supervised and neural network algorithms used in this study along with their hyperparameters are reported in Table 2. In the training process of each model, we employed the grid search method to identify the most suitable hyperparameters for the given dataset.

**Table 2:** Hyperparameter configuration of all supervised, and neural network models used for aneurysm rupture classification.

Model	Hyperparameters
Extreme Gradient Boosting (XGBoost)	booster = ‘gbtree’, eta=0.05, gamma=0, max_depth=4, learning_rate=0.05, subsample=0.5, colsample_bytree=0.5, alpha=1, tree_method=’hist’
Support Vector Machine (SVM)	C = 80, gamma = 0.002, kernel = ‘rbf’, probability = True
Stochastic Gradient Descent (SGD)	loss = ‘log_loss’, penalty=’l1’, max_iter = 10000, tol = 7e-5, alpha = 0.0002, eta0 = 0.01, learning_rate = ’adaptive’
K-Nearest Neighbors (KNN)	n_neighbors=17, weights=‘uniform’, p=1, algorithm=’auto’
Gaussian Process	kernel=1*RBF(2), optimizer=‘fmin_l_bfgs_b’, n_restarts_optimizer=0
Gaussian Naïve Bayes (GaussianNB)	var_smoothing = 1e-10
Decision Tree	criterion = ‘gini’, splitter=’random’, max_depth = 4, max_features = None, min_samples_split = 12, random_state = None
Bagging (SVM base estimator)	estimator = svm.SVC(C = 80, gamma = 0.002, probability = True, kernel = ‘rbf’), n_estimators = 100, bootstrap = True, max_samples = 0.9, max_features = 54
Bagging (KNN base estimator)	estimator = KNeighborsClassifier(n_neighbors = 5, weights = ‘uniform’, algorithm=’auto’), n_estimators = 100, bootstrap = True, max_samples = 0.9, max_features = 0.7
Random Forest (RF)	n_estimators = 80, criterion = ‘log_loss’, max_depth = 5, min_samples_leaf = 5, max_features = 0.5, min_samples_split = 20, bootstrap = True
AdaBoost (AdaBoost)	n_estimators=110, learning_rate=0.15
Gradient Boosting (GradientBoosting)	n_estimators=60, learning_rate=0.02, max_depth=4, min_samples_split=10
Histogram Gradient Boosting (HistGradientBoosting)	max_iter = 150, learning_rate = 0.03, loss = ‘log_loss’, max_leaf_nodes = 10, min_samples_leaf = 20
Multi-Layer Perceptron (MLP)	hidden_layer_sizes=(55,18,1), tol = 1e-6, batch_size = 28, max_iter = 2500, activation = ‘identity’, solver=’sgd’, alpha = 0.2, learning_rate_init = 0.00005, learning_rate = ’adaptive’
1D-CNN (Keras Sequential)	Architecture: Conv1D(64,3,‘relu’, input_shape=(55,1)) → Conv1D(64,5,’relu’) → Flatten → Dense(128,’relu’) → BatchNormalization → Dropout(0.2) → Dense(128,’relu’) → BatchNormalization → Dropout(0.4) → Dense(1,’sigmoid’); Training: optimizer = Adamax(learning_rate = 0.003), loss = ’binary_crossentropy’, EarlyStopping(monitor = ’val_loss’, patience = 25), epochs = 200, batch_size = 16, validation split via train_test_split(test_size = 0.12)

We evaluated model performance using the area under the receiver operating characteristic curve (ROC_AUC). We also computed accuracy, precision, recall (sensitivity), specificity, and F1-score from the confusion matrix. These metrics were evaluated on the validation partition of each fold during stratified 5-fold cross-validation within the training set following a stratified 90/10 data split, and the mean ± standard deviation across folds were reported. To further assess model generalizability and reliability, performance was subsequently evaluated on the independent 10% hold-out test set. Because the clinical cost of missing a high-risk (ruptured) case is high, recall was prioritized and, together with specificity, used to describe the operating behavior of each model. To examine model performance under a sensitivity-prioritized operating point, classification thresholds were tuned using five-fold cross-validated out-of-fold models to increase the average recall to 0.90 for the training set, and then the optimized thresholds were subsequently applied to the independent 10% test set without refitting the models. Predicted probabilities $\hat{p}$ were converted to class labels using a threshold τ:


$$\begin{array}{c} \hat{y}=\left\{ \begin{array}{c} \;if\;\hat{p}\geq \;\tau \\ \text{0}\;Otherwise \end{array}\right. \end{array}$$ (5)

Lowering τ increases recall by reducing false negatives (Default value is usually 0.5).

We computed permutation importance on the training data using repeated cross-validation with 10 repeats for the three best-performed models. For each feature, values were randomly permuted while keeping other variables fixed, and the resulting decrease in model ROC_AUC value was recorded. Mean importance and standard deviation were calculated across folds to assess both magnitude and stability of feature contribution. We presented the dominant features using radial plots. To this end, we first normalized permutation importance values within each model by dividing each feature’s mean importance by the total sum of absolute importance values, allowing the circular circumference to represent 100% of relative contribution and enabling comparison of dominant features against the full feature set.

To assess the contribution of different feature domains under a controlled modeling framework, three feature configurations were evaluated using the best-performing model architecture: clinical + morphological, clinical + hemodynamic, and combination of clinical + morphological + hemodynamic. Clinical variables were retained in all configurations as a common baseline. The same data partition and evaluation procedure were maintained across scenarios so that differences could be attributed to feature composition rather than model architecture.

To determine whether the dominant predictors alone could retain the performance of the full feature set, an additional model was trained using the best-performing model architecture and 5 dominant variable identified through the permutation importance. The performance and computational cost of this reduced-feature model were then compared with those of the full-feature model.

We implemented the machine-learning pipeline in Python. Classical machine-learning models and evaluation procedures were developed using scikit-learn, gradient boosting models were implemented using XGBoost, and deep-learning models were implemented using TensorFlow/Keras. Data preprocessing and analysis were performed using standard scientific computing libraries (NumPy and Pandas). All hyperparameters used in the final models are reported in Table 1 and Table 2 to ensure reproducibility.

## Results

Unsupervised clustering was used as an exploratory check of whether rupture status emerges as an intrinsic structure in the feature space. Fig 4 summarizes the results, showing that best-mapped accuracy ranged from 0.56 to 0.64 across the evaluated algorithms, with DBSCAN and Birch achieving the highest agreement (0.643), followed by Spectral (0.629) and Agglomerative clustering (0.616).

**Fig 4:**
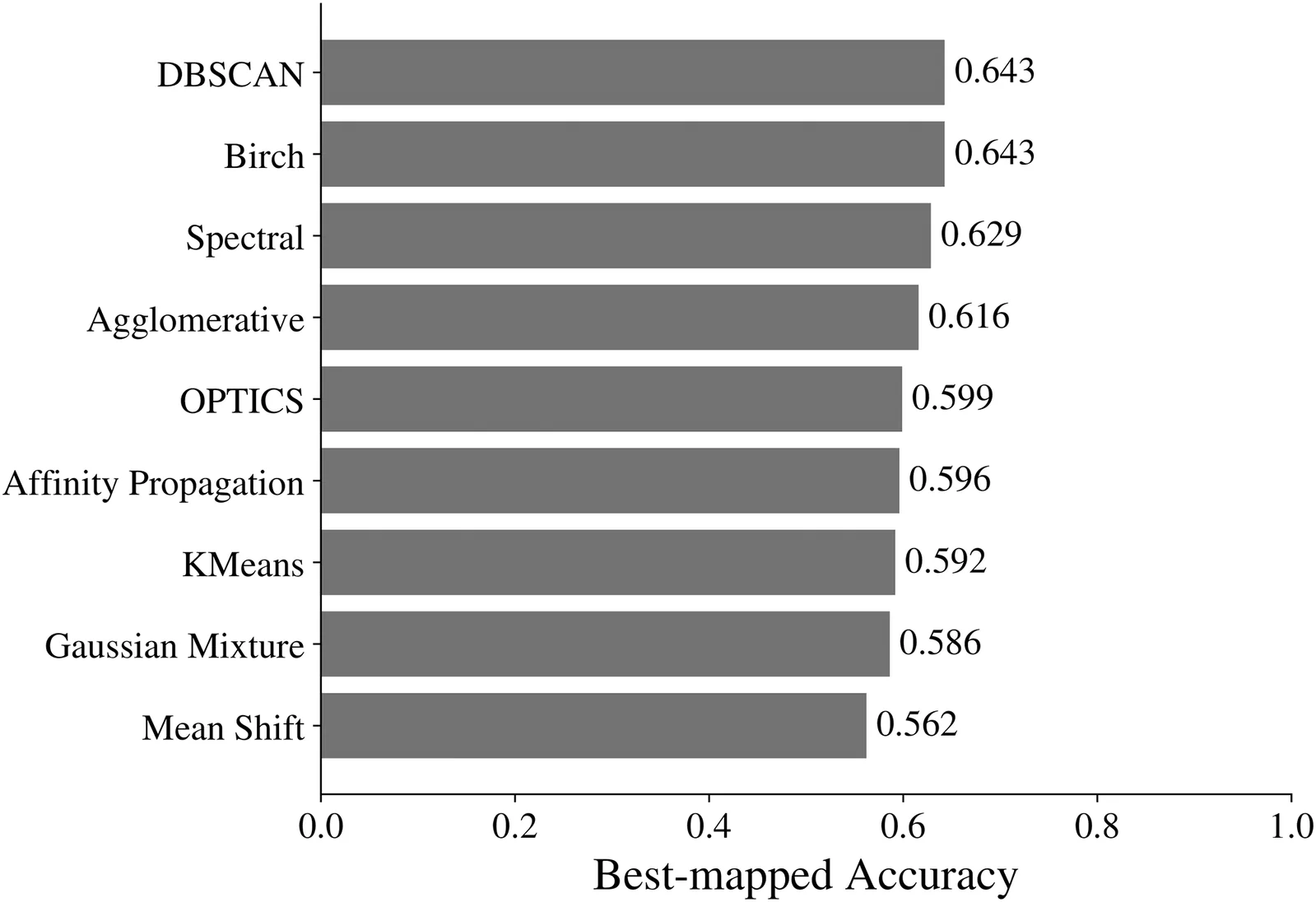
Comparison of unsupervised clustering algorithms. Evaluated using best-mapped accuracy, defined as the maximum classification agreement obtained after optimal permutation of cluster labels to true rupture labels.

Table 3 summarizes the cross-validated performance of the supervised classifiers included in the comparative analysis. Overall discrimination, as measured by mean ROC–AUC, was highest for SVM (0.817 ± 0.041), followed closely by Gaussian Process (0.814 ± 0.041) and BaggingSVM (0.813 ± 0.041). BaggingSVM achieved the highest mean precision (0.747 ± 0.051) and the highest specificity (0.907 ± 0.027), but with a comparatively lower recall (0.487 ± 0.103), indicating a more conservative classification tendency favoring true negatives. MLP also demonstrated strong overall performance (ROC–AUC 0.808 ± 0.043) with balanced precision (0.695 ± 0.052) and recall (0.605 ± 0.091).

**Table 3:** Five-fold cross-validation performance of all models on the 90% training set. Values are reported as mean ± standard deviation across folds.

Model	ROC_AUC	Accuracy	Precision	Recall	F1-score	Specificity
SVM	0.817 ± 0.041	0.744 ± 0.033	0.673 ± 0.062	0.566 ± 0.072	0.612 ± 0.053	0.844 ± 0.044
GaussianProcess	0.814 ± 0.041	0.750 ± 0.033	0.671 ± 0.045	0.597 ± 0.111	0.627 ± 0.069	0.836 ± 0.041
BaggingSVM	0.813 ± 0.041	0.757 ± 0.030	0.747 ± 0.051	0.487 ± 0.103	0.584 ± 0.072	0.907 ± 0.027
MLP	0.808 ± 0.043	0.765 ± 0.039	0.695 ± 0.052	0.605 ± 0.091	0.645 ± 0.071	0.853 ± 0.024
1D-CNN	0.799 ± 0.021	0.743 ± 0.026	0.662 ± 0.068	0.596 ± 0.089	0.621 ± 0.048	0.824 ± 0.055
XGBoost	0.791 ± 0.039	0.735 ± 0.035	0.621 ± 0.042	0.653 ± 0.083	0.636 ± 0.061	0.780 ± 0.013
SGD	0.790 ± 0.066	0.725 ± 0.038	0.592 ± 0.036	0.728 ± 0.100	0.652 ± 0.062	0.724 ± 0.009
RandomForest	0.784 ± 0.033	0.732 ± 0.032	0.610 ± 0.039	0.693 ± 0.059	0.648 ± 0.046	0.753 ± 0.027
HistGradientBoosting	0.781 ± 0.049	0.725 ± 0.042	0.636 ± 0.065	0.535 ± 0.088	0.580 ± 0.078	0.831 ± 0.024
KNN	0.781 ± 0.037	0.732 ± 0.036	0.690 ± 0.080	0.457 ± 0.063	0.548 ± 0.066	0.885 ± 0.033
AdaBoost	0.773 ± 0.044	0.738 ± 0.034	0.675 ± 0.053	0.513 ± 0.075	0.582 ± 0.065	0.863 ± 0.023
GradientBoosting	0.772 ± 0.038	0.717 ± 0.013	0.652 ± 0.026	0.452 ± 0.038	0.533 ± 0.030	0.866 ± 0.015
BaggingKNN	0.772 ± 0.036	0.700 ± 0.032	0.569 ± 0.042	0.689 ± 0.051	0.622 ± 0.038	0.707 ± 0.047
GaussianNB	0.741 ± 0.026	0.699 ± 0.033	0.572 ± 0.042	0.645 ± 0.080	0.604 ± 0.047	0.729 ± 0.055
DecisionTree	0.737 ± 0.036	0.711 ± 0.034	0.612 ± 0.057	0.526 ± 0.084	0.564 ± 0.064	0.814 ± 0.037

The 1D-CNN achieved a mean ROC–AUC of 0.799 ± 0.021, with recall of 0.596 ± 0.089 and F1-score of 0.621 ± 0.048, placing it within the upper mid-range of the evaluated models. XGBoost and Random Forest exhibited relatively balanced behavior, with recall values of 0.653 ± 0.083 and 0.693 ± 0.059, respectively, and ROC–AUC values around 0.79 and 0.78. SGD yielded the highest recall (0.728 ± 0.100) and the highest F1-score (0.652 ± 0.062), but with reduced precision (0.592 ± 0.036) and specificity (0.724 ± 0.009), reflecting a more liberal decision boundary.

Among the remaining models, KNN showed high specificity (0.885 ± 0.033) but substantially lower recall (0.457 ± 0.063), again suggesting conservative classification. AdaBoost and Gradient Boosting achieved moderate discrimination (ROC–AUC 0.773 ± 0.044 and 0.772 ± 0.038, respectively), with stronger specificity than recall. GaussianNB and Decision Tree exhibited the lowest overall discrimination (ROC–AUC 0.741 ± 0.026 and 0.737 ± 0.036), although their performance remained above random classification. Overall, the results indicate a trade-off between sensitivity and specificity across models, with SVM-based approaches achieving the strongest discrimination and ensemble methods offering more balanced recall–specificity profiles.

Fig 5 presents the performance of the 15 classifiers on the independent 10% test set. Among the evaluated models, BaggingSVM achieved the strongest discrimination on the independent test set (ROC–AUC = 0.783; PR–AUC = 0.643), followed by SVM (ROC–AUC = 0.752; PR–AUC = 0.555) and Gaussian Process (ROC–AUC = 0.748; PR–AUC = 0.540). KNN (0.738) and MLP (0.730) also demonstrated competitive discrimination, while the remaining models clustered between 0.67 and 0.72. Accuracy was highest for BaggingSVM (0.718), with SVM (0.704), Gaussian Process (0.690), and KNN (0.690) showing similar performance. Regarding precision, BaggingSVM reached the largest value (0.619), followed by SVM (0.591) and Gaussian Process (0.560). In contrast, recall (sensitivity) was highest for AdaBoost (0.800) and SGD (0.760), followed by DecisionTree (0.720) and KNN and GaussianNB (both 0.640), indicating that these models favored identifying positive cases, often at the expense of specificity. The highest F1-score was obtained by AdaBoost (0.606), followed by SGD (0.594) and KNN (0.593), demonstrating different trade-offs between precision and recall across models. Specificity was greatest for BaggingSVM (0.826), followed by SVM and HistGradientBoosting (both 0.804), reflecting stronger negative-class discrimination for these methods.

**Fig 5:**
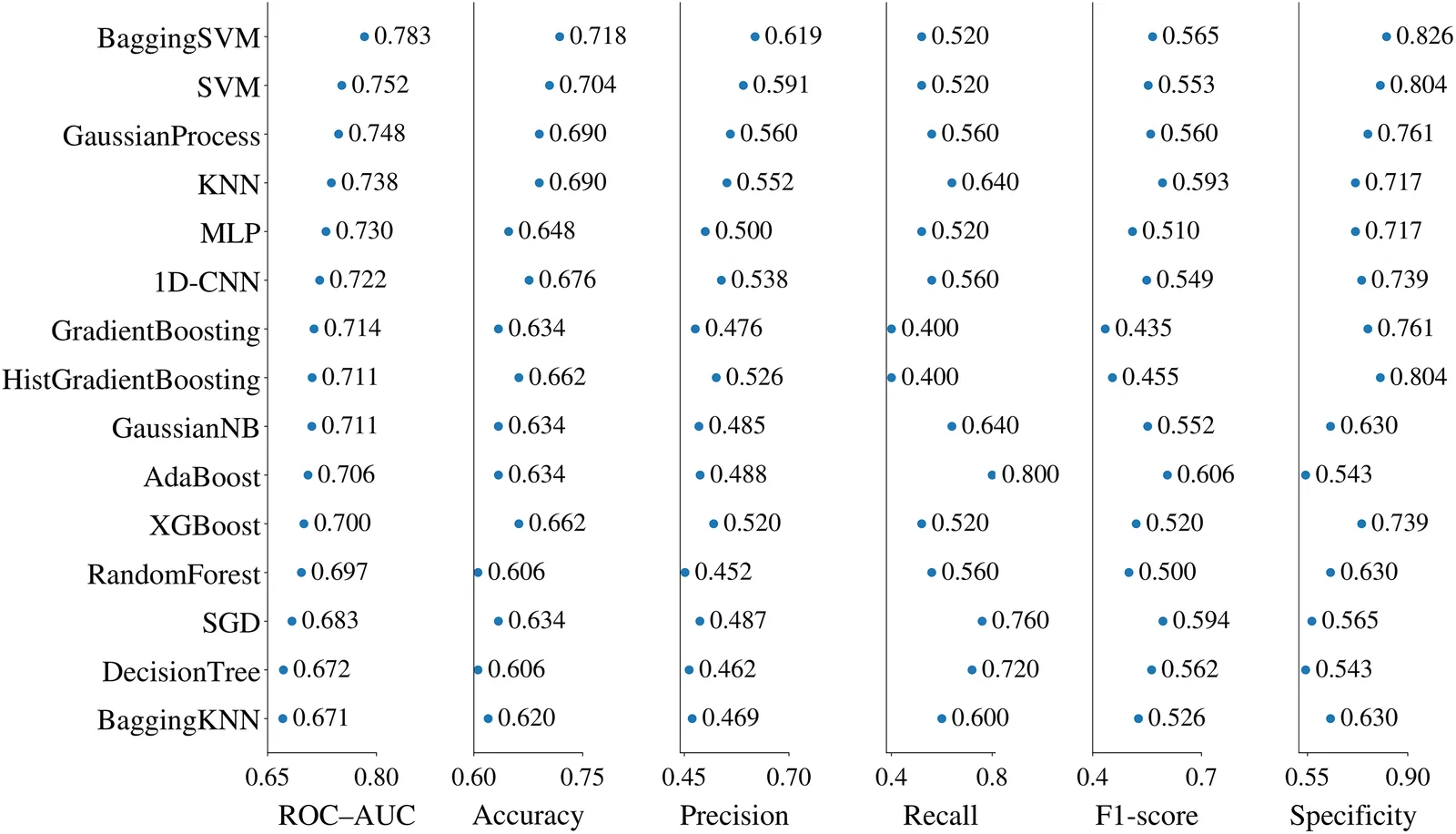
Performance of 15 supervised classification models evaluated on the independent 10% held-out test set. Each panel displays a different performance metric (ROC–AUC, Accuracy, Precision, Recall, F1-score, and Specificity). Points represent metric values for each model, ordered by ROC–AUC.

Overall, BaggingSVM showed the strongest overall discrimination and specificity on the independent test set. AdaBoost and SGD emphasized sensitivity and F1-score, whereas KNN balanced accuracy, recall, and F1-score with moderate specificity. These results highlight distinct decision tendencies across classifiers when evaluated on unseen data.

Fig 6 presents the performance of the three best-performing classifiers (BaggingSVM, SVM, and Gaussian Process) on the independent 10% test set before and after threshold adjustment. Using the default classification threshold of 0.50 (Fig 6 (a)), BaggingSVM achieved the highest overall discrimination (ROC–AUC = 0.78), followed by SVM (0.75) and Gaussian Process (0.75). In terms of accuracy, BaggingSVM performed best (0.72), while SVM and Gaussian Process achieved 0.70 and 0.69, respectively. Precision was also highest for BaggingSVM (0.62), compared to SVM (0.59) and Gaussian Process (0.56). Recall values were moderate, ranging from 0.52 (BaggingSVM and SVM) to 0.56 (Gaussian Process). Specificity was strongest for BaggingSVM (0.83), followed by SVM (0.80) and Gaussian Process (0.76). Overall, the default threshold favored balanced discrimination with relatively high specificity but moderate sensitivity. After threshold tuning to prioritize sensitivity (Fig 6 (b)), recall increased substantially across all models. BaggingSVM achieved a recall of 0.96, while both SVM and Gaussian Process reached 0.84. However, this improvement in sensitivity was accompanied by notable reductions in specificity, which declined to 0.48 for BaggingSVM and 0.43 for both SVM and Gaussian Process. Precision also decreased, particularly for SVM and Gaussian Process (both 0.45), while BaggingSVM maintained comparatively higher precision (0.50). Accuracy decreased accordingly (0.65 for BaggingSVM and 0.58 for the other two models). Importantly, ROC–AUC values remained unchanged, as expected, because threshold modification does not affect ranking-based discrimination.

**Fig 6:**
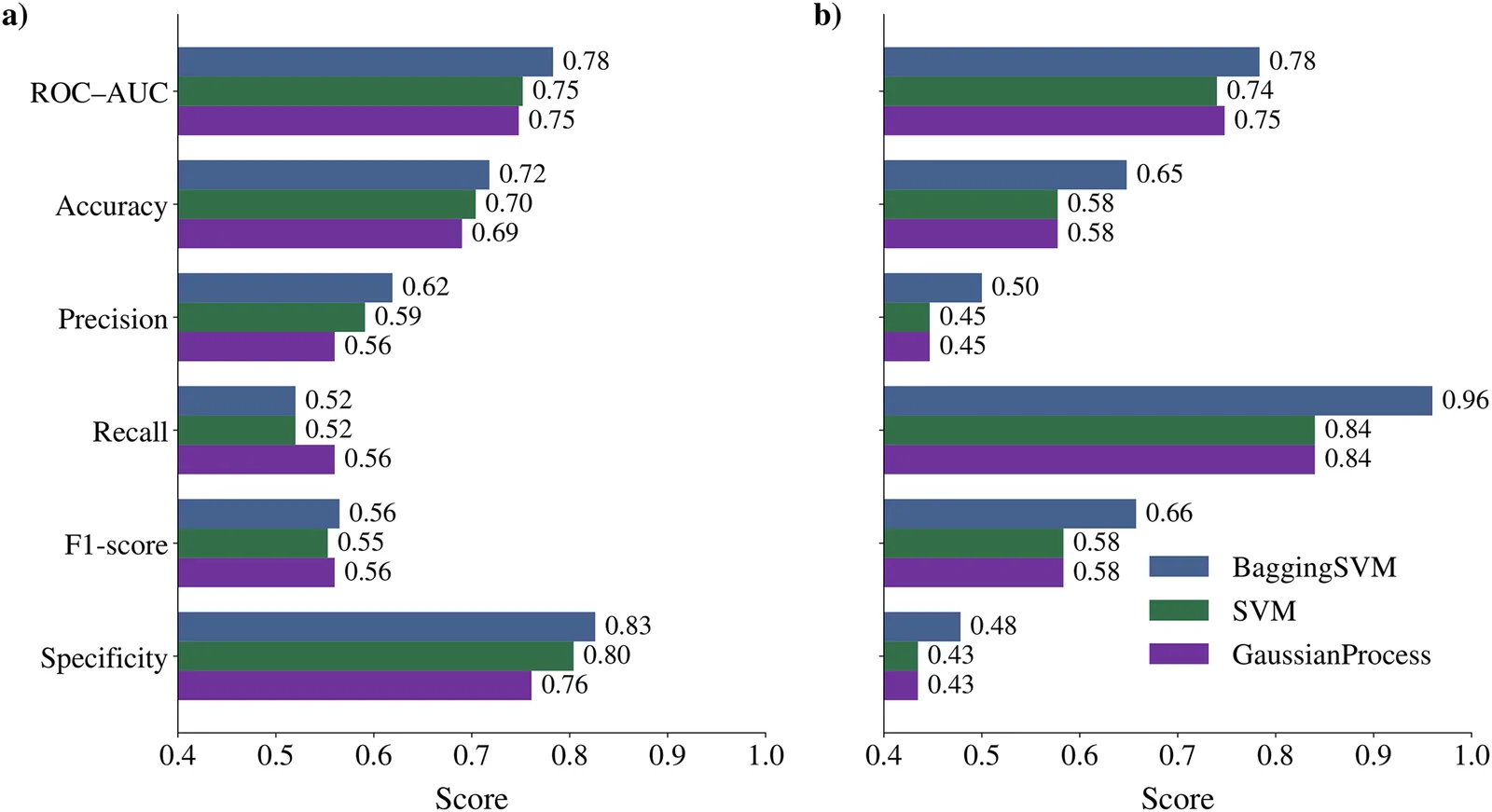
Top-three classifier performance on the independent test set before and after threshold tuning. (a) shows the original model performance using the default classification threshold of 0.50. (b) presents the performance after threshold adjustment.

Table 4 presents the permutation importance results (mean ± standard deviation) for all features across the three top-performing models: BaggingSVM, SVM, and Gaussian Process. Across models, a consistent pattern emerges in which irregularity (I), low shear area ratio (LSAR), Location, ellipticity index (EI), and aneurysm angle (MDNA) rank among the most influential input variables. For SVM and Gaussian Process, I is the highest-ranked feature, followed by LSAR and Location, indicating strong and stable contribution to model discrimination. In contrast, BaggingSVM assigns markedly higher importance to EI and LSAR, with substantially larger mean importance values compared to the other models. Beyond the top-ranked variables, importance values decline sharply, and most lower-ranked features exhibit near-zero or negative mean permutation importance, indicating minimal or no contribution to classification performance. Standard deviations are generally small relative to mean values for highly ranked features, suggesting stable importance across folds, while lower-ranked features display greater variability relative to their mean effect size. Overall, the results demonstrate strong agreement among models regarding the core important features, with differences primarily in magnitude rather than ranking structure.

**Table 4:** Permutation feature importance (mean ± standard deviation) for all input variables across the three best-performing classifiers. Importance values represent the average decrease in model performance (ROC_AUC) after random permutation of each feature. Features are ranked separately within each model based on mean importance.

Feature	BaggingSVM		SVM		Gaussian Process	
	Mean ± SD	Rank	Mean ± SD	Rank	Mean ± SD	Rank
EI	0.0584 ± 0.0224	1	0.0063 ± 0.0055	7	0.0071 ± 0.0063	6
LSAR	0.0552 ± 0.0115	2	0.0151 ± 0.0046	3	0.0187 ± 0.0045	2
I	0.0394 ± 0.0076	3	0.0459 ± 0.0154	1	0.0333 ± 0.0092	1
Location	0.0240 ± 0.0053	4	0.0235 ± 0.0071	2	0.0179 ± 0.0049	3
SR	0.0239 ± 0.0176	5	0.0034 ± 0.0053	10	0.0023 ± 0.0033	10
MDNA	0.0227 ± 0.0100	6	0.0131 ± 0.0081	4	0.0104 ± 0.0067	4
PL	0.0200 ± 0.0118	7	0.0003 ± 0.0037	35	0.0009 ± 0.0026	19
NWSS	0.0164 ± 0.0106	8	0.0016 ± 0.0037	16	0.0016 ± 0.0031	14
OSI	0.0137 ± 0.0124	9	−0.0007 ± 0.0028	48	−0.0001 ± 0.0024	28
ON	0.0136 ± 0.0060	10	0.0080 ± 0.0042	5	0.0088 ± 0.0064	5
IMDA	0.0113 ± 0.0082	11	0.0066 ± 0.0030	6	0.0063 ± 0.0026	7
MDPN	0.0096 ± 0.0085	12	0.0017 ± 0.0024	15	0.0001 ± 0.0019	25
DPVI	0.0095 ± 0.0098	13	0.0043 ± 0.0022	9	0.0037 ± 0.0041	8
IRR	0.0064 ± 0.0065	14	0.0020 ± 0.0044	13	0.0017 ± 0.0030	12
UI	0.0063 ± 0.0064	15	−0.0002 ± 0.0019	44	−0.0012 ± 0.0026	47
OVI	0.0057 ± 0.0041	16	0.0004 ± 0.0011	34	−0.0009 ± 0.0017	44
CP	0.0054 ± 0.0049	17	0.0025 ± 0.0017	11	0.0013 ± 0.0017	15
NPD	0.0045 ± 0.0034	18	0.0024 ± 0.0027	12	0.0017 ± 0.0026	11
FV	0.0041 ± 0.0112	19	0.0010 ± 0.0025	19	0.0003 ± 0.0040	22
MPH	0.0041 ± 0.0073	20	0.0006 ± 0.0023	30	−0.0004 ± 0.0014	30
Sex	0.0039 ± 0.0061	21	0.0047 ± 0.0063	8	0.0036 ± 0.0058	9
BR	0.0038 ± 0.0067	22	−0.0011 ± 0.0011	51	−0.0017 ± 0.0019	52
INA	0.0031 ± 0.0041	23	0.0006 ± 0.0014	31	0.0017 ± 0.0028	13
NR	0.0030 ± 0.0046	24	0.0003 ± 0.0011	36	0.0000 ± 0.0027	27
Age	0.0027 ± 0.0024	25	0.0009 ± 0.0014	22	−0.0009 ± 0.0018	43
IS	0.0027 ± 0.0042	26	0.0008 ± 0.0026	24	−0.0004 ± 0.0016	31
ND	0.0023 ± 0.0029	27	0.0009 ± 0.0024	23	0.0004 ± 0.0018	21
NDR	0.0022 ± 0.0031	28	−0.0009 ± 0.0012	50	−0.0008 ± 0.0013	42
EL	0.0017 ± 0.0049	29	0.0010 ± 0.0020	20	0.0006 ± 0.0021	20
IOR	0.0016 ± 0.0027	30	0.0019 ± 0.0039	14	0.0009 ± 0.0038	18
TT	0.0015 ± 0.0030	31	0.0010 ± 0.0017	21	0.0013 ± 0.0011	16
VOA	0.0012 ± 0.0013	32	−0.0000 ± 0.0013	41	−0.0015 ± 0.0011	50
AFI	0.0012 ± 0.0089	33	0.0006 ± 0.0041	29	−0.0004 ± 0.0043	32
NA	0.0011 ± 0.0020	34	0.0007 ± 0.0019	26	0.0001 ± 0.0015	24
DV	0.0011 ± 0.0012	35	0.0001 ± 0.0008	40	−0.0010 ± 0.0017	46
WSS	0.0009 ± 0.0028	36	0.0012 ± 0.0015	18	−0.0004 ± 0.0006	33
AR	0.0009 ± 0.0027	37	−0.0012 ± 0.0016	52	−0.0009 ± 0.0018	45
DA	0.0008 ± 0.0017	38	0.0004 ± 0.0016	32	−0.0013 ± 0.0015	48
IR	0.0007 ± 0.0030	39	0.0006 ± 0.0026	28	0.0002 ± 0.0024	23
PR	0.0005 ± 0.0015	40	−0.0001 ± 0.0023	42	−0.0006 ± 0.0032	36
MD	0.0004 ± 0.0053	41	−0.0008 ± 0.0018	49	−0.0022 ± 0.0015	54
PLC	0.0004 ± 0.0012	42	0.0008 ± 0.0010	25	0.0010 ± 0.0019	17
NC	0.0003 ± 0.0025	43	0.0001 ± 0.0018	39	−0.0005 ± 0.0017	34
FS	0.0003 ± 0.0015	44	0.0012 ± 0.0015	17	−0.0008 ± 0.0008	41
FC	0.0002 ± 0.0035	45	−0.0002 ± 0.0028	43	−0.0001 ± 0.0023	29
WSSD	0.0001 ± 0.0032	46	−0.0020 ± 0.0031	53	−0.0007 ± 0.0017	39
TAWSS	−0.0004 ± 0.0038	47	0.0003 ± 0.0014	37	−0.0006 ± 0.0006	35
NSI	−0.0007 ± 0.0031	48	0.0004 ± 0.0018	33	−0.0007 ± 0.0014	37
SF	−0.0008 ± 0.0039	49	−0.0003 ± 0.0015	45	−0.0013 ± 0.0014	49
ISR	−0.0013 ± 0.0011	50	0.0007 ± 0.0028	27	−0.0008 ± 0.0009	40
CPH	−0.0014 ± 0.0030	51	−0.0005 ± 0.0016	47	−0.0019 ± 0.0010	53
WSSG	−0.0015 ± 0.0103	52	−0.0030 ± 0.0042	55	−0.0016 ± 0.0023	51
RRT	−0.0017 ± 0.0023	53	−0.0003 ± 0.0011	46	0.0001 ± 0.0005	26
LB	−0.0017 ± 0.0018	54	0.0002 ± 0.0019	38	−0.0007 ± 0.0021	38
COD	−0.0045 ± 0.0030	55	−0.0023 ± 0.0018	54	−0.0038 ± 0.0035	55

Fig 7 illustrates the relative distribution of feature importance across the three best-performed classifiers. In all models, classification contribution is highly concentrated within a small subset of features, while the majority contribute minimally. For SVM and Gaussian Process, the feature *I* dominates the importance distribution, occupying the largest proportion of the circumference, followed by LSAR and Location-related metrics. In contrast, BaggingSVM shows a more distributed but still concentrated pattern, with EI and LSAR contributing substantially larger proportions relative to other features. The remaining variables form progressively smaller segments, confirming that most variables have negligible impact on ROC–AUC when permuted. Once again, the visualization demonstrates strong agreement across models regarding the primary important features, while highlighting differences in the magnitude and concentration of importance.

**Fig 7:**
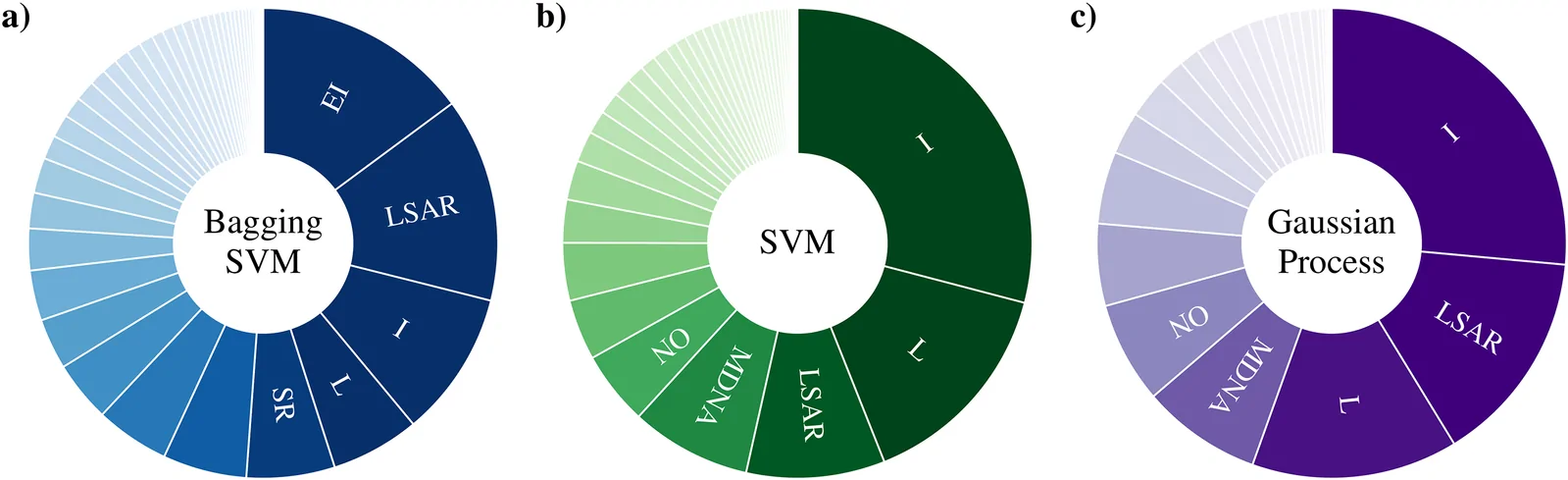
Radial plots of normalized permutation feature importance for the three top-performing classifiers. (a) BaggingSVM, (b) SVM, and (c) Gaussian Process. The circumference of each donut chart represents the total normalized importance (100%), with segment sizes proportional to each feature’s relative contribution based on mean permutation importance (ROC–AUC decrease). The five dominant features are labeled, while remaining features are shown in lighter shades to illustrate their comparatively smaller contributions.

Fig 8 presents the feature importance comparison across clinical+morphological features, clinical+hemodynamical features, and combined scenarios using the same Bagging SVM model. The classification performance is the highest when combining feature domains, with an AUC of 0.78. For clinical+morphological features and clinical+hemodynamical features, AUC slightly decreased to 0.76 and 0.71, respectively. In the clinical+morphological features scenario (Fig 8 (a)), ellipticity index (EI) and irregularity (I) are the dominant predictors, followed by location, and other geometric descriptors. In the clinical+hemodynamical features scenario (Fig 8 (b)), low shear area ratio (LSAR) is the most influential variable, followed by location, and additional flow-related metrics such as wall shear stress (WSS) and oscillatory shear index (OSI). In the combined scenario (Fig 8 (c)), EI and LSAR remain the leading predictors, and location remains consistently relevant across all scenarios. These results indicate that combining morphological and hemodynamic features enhances classification performance and alters the relative contribution of individual predictors.

**Fig 8:**
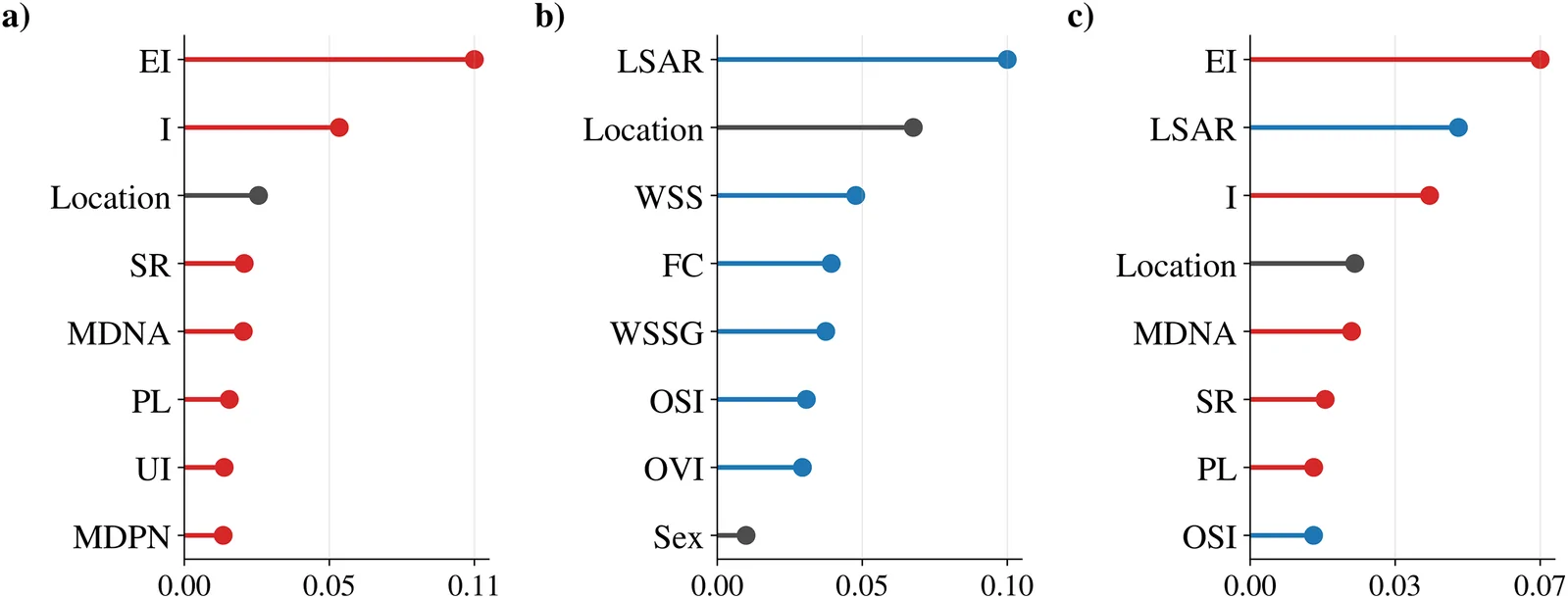
Dominant features across three feature scenarios using Bagging SVM. (a) clinical+morphological features, (b) clinical+hemodynamical features, and (c) combined feature set. Clinical variables are included in all scenarios. Each panel shows the top predictors ranked by normalized permutation importance (sum = 1 within each panel). Horizontal lines represent relative feature importance, and colors indicate feature type: morphological (red), hemodynamic (blue), and clinical (gray). The comparison highlights how the relative importance of predictors changes when morphological and hemodynamic descriptors are evaluated jointly under the same model.

A reduced-feature model was trained using BaggingSVM with only the five dominant features as input variables (EI, LSAR, I, aneurysm location, and MDNA). The model achieved a mean cross-validation ROC–AUC of 0.789 ± 0.054, compared with 0.813 ± 0.041 for the full 55-feature model. On the independent test set, ROC–AUC decreased from 0.783 to 0.717, while accuracy decreased from 0.718 to 0.662 and recall remained at 0.44. Thus, although the five dominant predictors captured a substantial portion of the discriminatory information, they were insufficient to reproduce the performance of the complete feature set. Reducing the input space from 55 to 5 predictors decreased the dimensionality by approximately 91% and reduced machine-learning training time by approximately 52%, while simultaneously improving model simplicity and interpretability.

In addition to categorical classification, the CNN provides probabilistic outputs between 0 and 1, which enables a more nuanced interpretation of rupture likelihood. Aneurysms with output values closer to 1 are more likely to be ruptured, whereas values closer to 0 indicate a higher likelihood of being unruptured. Importantly, outputs near the decision threshold of 0.50 may represent intermediate-risk cases where the model is less confident. For the four example aneurysms shown in Fig 2, the CNN rated rupture probabilities of 20.94% for Fig 2 (a), 47.46% for Fig 2 (b), 10.98% for Fig 2 (c), and 60.66% for Fig 2 (d). Using a 50% threshold, only aneurysm (d) is classified as ruptured, which matches the true label for this example set.

## Discussion

This study provides a comprehensive evaluation of machine learning approaches for classifying rupture status in cerebral aneurysms using an integrated set of clinical, morphological, and hemodynamic features. By comparing a wide range of supervised and unsupervised algorithms and incorporating physiologically informed hemodynamic simulations, the results provide insights into both the classification performance of different modeling strategies and the underlying factors associated with aneurysm rupture.

The unsupervised clustering results (Fig 4) suggest that rupture status does not emerge as a strongly separable structure in the feature space when label information is absent. Best-mapped clustering accuracy ranged from approximately 0.56 to 0.64 across the evaluated methods, indicating only modest alignment between cluster assignments and rupture labels. This observation implies that ruptured and unruptured aneurysms are not naturally separated by the feature distribution alone and that supervised learning methods, which explicitly incorporate rupture labels during training, are more suitable for reliable rupture discrimination.

Among the supervised classifiers, several models demonstrated competitive performance (Fig 5), with BaggingSVM, SVM, and Gaussian Process achieving the strongest discrimination on the independent test set. BaggingSVM achieved the highest ROC–AUC (0.78) and accuracy (0.72), while also maintaining the highest specificity among evaluated models. In contrast, AdaBoost and SGD emphasized sensitivity, achieving the highest recall values but with lower specificity. These differences highlight an inherent trade-off between sensitivity and specificity across classifiers, reflecting distinct decision boundaries and classification tendencies. In clinical applications such as aneurysm rupture classification, this trade-off is particularly important because missing a potentially ruptured aneurysm (false negative) may have more severe consequences than incorrectly flagging a stable aneurysm. Threshold adjustment further demonstrated this trade-off. Lowering the classification threshold increased recall substantially (Fig 6), allowing models to detect a greater proportion of ruptured aneurysms, but this improvement came at the cost of reduced specificity and increased false-positive classifications. Such behavior is expected in probabilistic classifiers and emphasizes the need to select operating thresholds that reflect the clinical priorities of screening versus confirmatory diagnosis. Semi-supervised methods (self-training and label spreading algorithms) were explored by treating them like supervised methods but did not provide additional benefit in the fully labeled setting and were therefore not included in the final comparative analysis. We also performed pairwise statistical comparisons of ROC–AUC among the three top-performing classifiers using a paired bootstrap procedure applied to predictions from the same independent test cases. No statistically significant differences were identified. Specifically, the comparison between BaggingSVM and SVM yielded a Holm-adjusted p-value of 0.391, BaggingSVM versus Gaussian Process yielded p = 0.672, and SVM versus Gaussian Process yielded p = 0.871. These results indicate that, although BaggingSVM achieved the highest observed ROC–AUC, its performance was not statistically different from that of SVM or Gaussian Process on the independent test set. This supports our descriptive interpretation of the model rankings rather than a claim of statistical superiority.

Permutation feature importance analysis revealed a consistent pattern across the three best-performing classifiers. Irregularity (I), low shear area ratio (LSAR), aneurysm location, ellipticity index (EI), and aneurysm angle (MDNA) emerged as the most influential variables of rupture status (Table 4). Although the models differed slightly in the magnitude of importance assigned to each feature, their ranking structure was largely consistent. These findings suggest that rupture classification is driven primarily by a small subset of variables rather than the full set of 55 input features. Fig 7 further supports this interpretation by showing that a substantial share of the total normalized importance is concentrated in only a few morphological and hemodynamic features.

A key finding of this study is when evaluated under a consistent modeling framework, the integration of morphological and hemodynamic features leads to both improved classification performance (highest AUC by combining all features) and a redistribution of feature importance (Fig 8). Unlike previous comparisons based on separate models, this analysis isolates the effect of feature domain by using the same Bagging SVM architecture across all scenarios. The results show that certain predictors, such as ellipticity index (EI) and low shear area ratio (LSAR), remain strongly influential regardless of feature configuration, suggesting that they capture fundamental aspects of aneurysm instability. In contrast, other variables exhibit reduced relative importance in the combined setting, indicating potential overlap or redundancy with other descriptors. This shift suggests that the combined model does not simply aggregate information but instead balances competing signals from different feature domains. The observed patterns are consistent with prior clinical and computational evidence linking geometric irregularity, shape deformation, and low-shear regions to aneurysm instability, wall remodeling, and rupture-related processes [12,15,16,46–50], and further demonstrate that a unified framework provides a more refined interpretation of their joint contribution.

The reduced-feature analysis further clarified the relationship between feature importance and model performance. Although EI, LSAR, aneurysm location, irregularity, and MDNA consistently emerged as the strongest individual predictors, restricting the BaggingSVM model to these five variables reduced independent test ROC–AUC from 0.774 to 0.717. This indicates that dominance in permutation importance does not imply that the remaining variables are collectively uninformative. Rather, lower-ranked predictors appear to provide additional complementary information that improves discrimination when considered jointly. The five-feature model substantially reduced dimensionality and machine-learning training time, but this computational advantage should be interpreted cautiously because LSAR remains CFD-derived and therefore still requires the underlying flow simulations. Consequently, the full feature set provided a better balance of discrimination and information content in the present dataset, whereas the reduced model offered greater simplicity at the expense of predictive performance.

An additional contribution of this work lies in the use of a non-Newtonian blood rheology model during the computational fluid dynamics simulations used to derive hemodynamic features. Many previous machine learning studies on aneurysm rupture classification rely on Newtonian approximations of blood viscosity, which may overestimate wall shear stress in small cerebral arteries. By employing the Carreau–Yasuda model, this study accounts for the shear-thinning behavior of blood and provides more physiologically realistic hemodynamic estimates [22]. As a result, features such as normalized wall shear stress and turnover time are likely captured with greater fidelity, potentially improving the reliability of the extracted hemodynamic variables [51].

Compared with previous machine learning studies on aneurysm rupture classification, the present work offers several methodological advantages. Earlier studies often relied on smaller datasets, single feature categories, or limited model comparisons. For example, random forest–based approaches [40] and models relying solely on morphological or hemodynamic parameters typically reported AUC values between approximately 0.78 and 0.81 [39]. By integrating morphological, clinical, and hemodynamic variables and evaluating a broad range of machine learning algorithms, the present analysis provides a more comprehensive perspective on the assessment landscape of aneurysm rupture risk. The use of a large multi-center dataset containing more than 700 aneurysm geometries increases the diversity of the analyzed cohort, although cross-center generalizability remains to be independently validated.

The probabilistic outputs produced by the convolutional neural network also provide an additional perspective on rupture risk assessment. Rather than limiting the result to a binary label, the CNN generates continuous probability estimates that may allow clinicians to interpret rupture risk along a spectrum of model-estimated rupture likelihood. Such probabilistic outputs may be particularly valuable in borderline or ambiguous cases where aneurysms exhibit intermediate characteristics. In time-sensitive clinical settings, these probabilistic estimates may help physicians interpret the relative strength of model confidence and support more nuanced treatment prioritization, even if the final decision must still be made in conjunction with clinical judgment and other diagnostic information.

Despite these strengths, several limitations should be acknowledged. A key limitation of this study is that the proposed framework performs retrospective rupture status classification using aneurysms already labeled as ruptured or unruptured. Therefore, it should not be interpreted as a prospective model for predicting future rupture events in currently unruptured aneurysms. Next, the dataset exhibits class imbalance and demographic imbalance, with a larger proportion of female patients and unruptured aneurysms (Fig 1). In addition, the four contributing datasets differ substantially in sample size and in the distributions of aneurysm location, sex, age, and rupture status. These differences limited the suitability of leave-one-center-out validation, because holding out an entire center would have produced test sets with markedly different case compositions and sample sizes. We therefore used stratified sampling and cross-validation to preserve the rupture-status distribution while maintaining an independent test set. Although permutation importance provided interpretable global estimates of feature relevance for the classical machine-learning models, interpretation of the CNN is more challenging because its predictions are generated through nonlinear and distributed interactions across multiple hidden representations. High classification performance alone does not indicate which input variables drive an individual prediction or how strongly they contribute. Explainability approaches such as integrated gradients, or other neural-network attribution methods could therefore be incorporated in future studies to provide global and case-specific explanations of CNN predictions. Such analyses may improve model transparency and facilitate clinical interpretation, although attribution values should be interpreted as explanations of model behavior rather than causal effects. The CFD-derived predictors are also subject to uncertainties introduced at several stages of the computational workflow. Mesh independence was evaluated at least five geometries, these cases were not prospectively stratified to represent the full range of aneurysm sizes, locations, and morphological complexity. In addition, patient-specific flow measurements were unavailable, and standardized pulsatile inlet waveforms were scaled using literature-derived values based on available patient characteristics. These assumptions may affect the magnitude and spatial distribution of CFD-derived variables, particularly wall-shear- and residence-time-related metrics, and may consequently propagate uncertainty into machine-learning performance and feature-importance estimates. Accordingly, the hemodynamic predictors should be interpreted as standardized computational estimates rather than fully individualized physiological measurements. Future studies should assess the robustness of these findings using broader mesh-sensitivity analyses, independently acquired cohorts, and patient-specific boundary conditions.

Overall, this study adds to the existing literature by moving beyond domain-specific analyses of cerebral aneurysm rupture status and evaluating clinical, morphological, and hemodynamic predictors within a single machine-learning framework. This unified approach makes it possible to identify which variables remain consistently informative when all feature domains are considered together, rather than interpreting morphology- and flow-based factors in isolation. The comparison across feature scenarios further showed that combining morphological and hemodynamic descriptors improved model performance compared with using either feature domain alone. In this setting, ellipticity index, low shear area ratio, aneurysm location, irregularity, and aneurysm angle emerged as repeatedly influential predictors, suggesting that rupture status classification is driven by a compact cross-domain subset of descriptors rather than by the full high-dimensional feature space. In addition to comparing multiple learning strategies under a common framework, the study also highlights the potential value of probabilistic model outputs for clinical interpretation. Future work should validate these findings in broader multi-center datasets, incorporate more clinically detailed patient information, and further improve interpretability to support eventual decision-support applications.

## Supporting information

S1 Fig WSS measured at the systolic peak for a sample vessel, including values across the aneurysm dome and its local average. (TIF)

S2 Fig NWSS measured at the systolic peak for a sample vessel, including values across the aneurysm dome and its local average. (TIF)

S3 Fig WSSG measured at the systolic peak for a sample vessel, including values across the aneurysm dome and its local average. (TIF)

S4 Fig WSSD measured at the systolic peak for a sample vessel, including values across the aneurysm dome and its local average. (TIF)

S5 Fig WSS at peak systole in a sample vessel, showing the aneurysm dome, mean parent-vessel WSS, and the aneurysm-dome region where WSS is below 10% of mean parent-vessel WSS. (TIF)

S6 Fig OSI values for the parent vessel, aneurysm dome, and the dome’s average, integrated over one cardiac cycle. (TIF)

S7 Fig TAWSS values for the parent vessel, aneurysm dome, and mean aneurysm-dome value over one cardiac cycle. (TIF)

S8 Fig AFI values for the parent vessel, aneurysm dome, and the dome’s average, evaluated at the systolic peak. (TIF)

S9 Fig RRT values for the parent vessel, aneurysm dome, and the dome’s average, evaluated at the systolic peak. (TIF)

S10 Fig Velocity vectors at the neck region during the systolic peak. (a) inlet and (b) outlet flow.
(TIF)

S11 Fig OVI values across multiple cross-sectional areas, integrated over one cardiac cycle. (TIF)

S1 Movie Streamlines illustrating flow stability and complexity throughout the cardiac cycle. (MP4)

S1 Appendix Hemodynamic and morphological feature definitions and calculation methods. (DOCX)
