# Supplementary material for: An integrated machine learning framework for cerebral aneurysm rupture status classification using clinical, morphological, and hemodynamic features
Source: PLoS One. 2026 Oct 30;21(10):e0360923. doi: 10.1371/journal.pone.0360923 (PMC13722296; doi:10.1371/journal.pone.0360923)
Supplement: Supplementary file 1 [file pone.0360923.s013.docx]

Hemodynamic and morphological feature definitions

S1 Appendix. Definitions and calculation methods for the features used in cerebral aneurysm rupture status classification. Corresponding visualizations are provided in S1–S11 Figs and S1 Movie.

# Hemodynamic features

**1.** **Wall Shear Stress (WSS):** Shear force per unit area exerted by blood flow on the vessel wall.

$$WSS= \mu\frac{\partial\vec{V}}{\partial\vec{n}}$$

where $\mu$ is dynamic viscosity of the blood, $\vec{V}$ is the velocity and $\vec{n}$ is the normal vector to the vessel wall.

**2. Normalized wall shear stress (NWSS):** WSS normalized by the parent artery's average WSS.

$$NWSS= \frac{{WSS}_{Aneurysm}}{{WSS}_{Parent Vessel}}$$

**3. Wall Shear Stress Gradient (WSSG):** Spatial gradient of WSS magnitude.

$$WSSG=\frac{\partial|WSS|}{\partial x}\vec{i}+\frac{\partial|WSS|}{\partial y}\vec{j}+\frac{\partial|WSS|}{\partial z}\vec{w}$$

**4. Wall shear stress divergence (WSSD):** Divergence of the WSS vector field.

$$WSSD=\frac{\partial\vec{{WSS}_{x}}}{\partial x}+\frac{\partial\vec{{WSS}_{y}}}{\partial y}+\frac{\partial\vec{{WSS}_{z}}}{\partial z}$$

**5. Low shear area ratio (LSAR):** The fraction of the saccular aneurysm area at peak systole where WSS is below 10% of the parent artery’s WSS.

$$LSAR=\frac{Area where WSS< 0.1\times{WSS}_{Parent}}{Dome Area}$$

**6. Oscillatory shear index (OSI):** Measures directional changes in WSS over the cardiac cycle.

$$OSI=\frac{1}{2}\left( 1-\frac{|\int_{0}^{T} {WSS}_{i}dt|}{\int_{0}^{T} {|WSS}_{i}|dt} \right)$$

where T is the duration of cardiac cycle.

**7. Time-Averaged Wall Shear Stress (TAWSS):** Computes the time-integrated WSS magnitude at each node over the cardiac cycle.

$$TAWSS=\frac{1}{T}\int_{0}^{T} {|WSS}_{i}|dt$$

**8. Aneurysm formation indicator (AFI):** Indicates alignment between instantaneous and average WSS vectors.

$$AFI=\frac{\vec{{WSS}_{i}}\cdot\vec{TAWSS}_{i}}{\left| {WSS}_{i} \right| \left| {TAWSS}_{i} \right|}$$

**9. Relative residence time (RRT):** Reflects low-magnitude, oscillatory flow regions.

$$RRT=\left[ \left( 1-2\times OSI \right)|WSS| \right]^{-1}$$

**10. Normalized pressure difference (NPD):** The difference between the peak pressure (accounting for both spatial and temporal variations) and the mean pressure, normalized by the inlet dynamic pressure at the aneurysm neck.

$$NPD=\frac{P_{max}-P_{avg}}{\frac{1}{2}\rho v_{in}^{2}}$$

where $P$ is static pressure and $v_{in}$ is the inlet velocity at the neck region.

**11. Pressure loss coefficient (PLC):** The drop in total (dynamic + static) pressure at the aneurysm neck, normalized by the inlet dynamic pressure at peak systole.

$$PLC=\frac{\left( \frac{1}{2}\rho v_{in}^{2}+P_{in} \right)-\left( \frac{1}{2}\rho v_{out}^{2}+P_{out} \right)}{\frac{1}{2}\rho v_{in}^{2}}$$

where $v_{out}$ is the outlet velocity at the neck region.

**12. Energy loss (EL):** The energy loss at the aneurysm neck caused by vortex formation and dome friction at peak systole.

$$EL=\frac{v_{in}A_{in}\left\{ \left( \frac{1}{2}\rho v_{in}^{2}+P_{in} \right)-\left( \frac{1}{2}\rho v_{out}^{2}+P_{out} \right) \right\}}{DV}$$

where $A_{in}$ is the inlet section area at the neck and $DV$ is the volume of the dome or saccular region.

**13. Turnover time (TT):** Time needed for blood to pass through the aneurysm.

$$TT=\frac{DV}{v_{in}A_{in}}$$

**14. flow velocity (FV):** Average velocity within the aneurysm dome at systolic peak.

**15. oscillatory velocity index (OVI):** Quantifies flow directional oscillations.

$$OVI=\frac{1}{2}\left( 1-\frac{|\int_{0}^{T} v_{i}dt|}{\int_{0}^{T} {|v}_{i}|dt} \right)$$

**16. Flow complexity (FC):** Qualitative indicator of flow divisions, separations, or vortex structures.

**17. Flow stability (FS):** Qualitative indicator of time-varying flow pattern changes within the aneurysm.

Among all hemodynamic parameters, **FS** and **FC** were encoded as binary values for input into the ML models, visually labeled by MA for consistency.

Further details of the governing equations, constitutive model parameters, blood-flow simulation setup, grid independence study, and hemodynamic metrics are provided by Zakeri et al. [1].

# Morphological features

Table A. Morphological features and their definitions.

| Parameters | Definition |
| --- | --- |
| Dome Volume (DV) | Insider volume of the saccular |
| Dome Area (DA) | Outer area of the saccular |
| Maximum Diameter (MD) | Maximum length between two distinct points on the saccular |
| Central Perpendicular Height (CPH) | Perpendicular height from the center of the neck |
| Neck Diameter (ND) | Diameter of the neck parallel to the blood flow |
| Neck Area (NA) | Area of the neck |
| Diameter of the Parent Vessel at the Inlet (DPVI) | Diameter of the parent vessel before the aneurysm |
| Maximum Perpendicular Height (MPH) | Maximum perpendicular height from the neck plane |
| Inflow Angle (IMDA) | Angle between the inlet flow and the MD |
| Aneurysm Angle (MDNA) | Angle between the neck and the MD |
| Neck Angle (INA) | Angle between the inlet flow and the neck |
| Projection Length (PL) | Length of the saccular shadow on the parent vessel |
| Maximum Diameter Parallel to the Neck (MDPN) | Distance between the neck and a parallel cut-plane of the saccular with the largest area |
| Lateral or Bifurcation (LB) Status | Location of the aneurysm in the parent vessel |
| Irregularity (I) | Deviation from the balloon shape |
| Aspect Ratio (AR) | $CPH / ND$ |
| Bottleneck Ratio (BR) | $MD / ND$ |
| Neck Ratio (NR) | $ND / DPVI$ |
| Size Ratio (SR) | $MD / DPVI$ |
| Dome to Neck Ratio (DNR) | $DA / NA$ |
| Shape Factor (SF) | ${DA/DV}^{2/3}$ |
| Volume to the Ostium Area (VOA) | $DV/NA$ |
| Ellipticity Index^*^ (EI) | $1-\left( 18\pi\right)^{\frac{1}{3}}{DV}_{CH}^{\frac{2}{3}}/{DA}_{CH}$ |
| Nonsphericity Shape Index (NSI) | $1-\left( 18\pi\right)^{\frac{1}{3}}{DV}^{\frac{2}{3}}/DA$ |
| Undulation Index^*^ (UI) | $1-DV/{DV}_{CH}$ |
| Projection Ratio (PR) | $MPH/DPVI$ |
| Conicity Parameter (CP) | $0.5-MDPN/DPVI$ |
| Neck Circumference (NC) | Perimeter of the neck |
| Ideal Roundness (IR) | $\sqrt{NA/\pi}$ |
| Ideal Roundness Ratio (IRR) | $2\pi(IR)/NC$ |
| Ideal Sphericity (IS) | $\sqrt[3]{3(DV)/4\pi}$ |
| Ideal Sphericity Ratio (ISR) | $4\pi\left( IS \right)^{2}/(DA+NA)$ |
| Outflow Number (ON) | Number of outlets from the saccular |
| Cumulative Outlet Diameter (COD) | Total diameter of all outlets from the saccular |
| Inlet to Outlet(s) Ratio (IOR) | $DPVI/COD$ |

* ${DV}_{CH}$ and ${DA}_{CH}$ are the volume and outer area of the convex hull of the aneurysm.

Among all morphology parameters, **I** and **LB** were encoded as binary values for input into the ML models, visually labeled by MZ for consistency.

Further details of morphological feature definitions and calculations are provided by Zakeri et al. [2].

# References

1. Zakeri M, Aziznia M, Atef A, Jafari A. Hemodynamic predictors of cerebral aneurysm rupture: A machine learning approach. Phys Fluids. 2024;36(9):097112. doi: 10.1063/5.0224289.

2. Zakeri M, Atef A, Aziznia M, Jafari A. A comprehensive investigation of morphological features responsible for cerebral aneurysm rupture using machine learning. Sci Rep. 2024;14:15777. doi: 10.1038/s41598-024-66840-1.
